# 4,4′-Methylenebis{*N*-[(*E*)-quinolin-2-yl­methylidene]aniline}

**DOI:** 10.1107/S1600536811016011

**Published:** 2011-05-07

**Authors:** Daoud Djamel, Douadi Tahar, Haffar Djahida, Hammani Hanane, Chafaa Salah

**Affiliations:** a Laboratoire d’Électrochimie des Matériaux Moléculaires et Complexes, (LEMMC), Département de Génie des Procèdes Faculté de Technologie, Université Ferhat Abbas, Setif 19000, Algeria

## Abstract

The title compound, C_33_H_24_N_4_, was prepared by the reaction of a bifunctional aromatic diamine (4,4′-diamino­diphenyl­methane) and an aldehyde (quinoline-2-carboxaldhyde). The mol­ecule consists of two nearly planar (or r.m.s. deviation = 0.017 Å) 4-methyl-*N*-[(*E*)-quinolin-2-yl­methyl­idene]aniline moieties, which are linked by the methyl­ene group. The angle between the mean planes of the two benzene rings connected to the methyl­ene group is 77.86 (11)°.

## Related literature

For the biological and pharmacological activity of quinolines and their derivatives, see: Kidwai *et al.* (2000 ▶); Souza (2005 ▶); Musiol *et al.* (2006 ▶); Gómez-Barrio *et al.* (2006 ▶); Vinsova *et al.* (2008 ▶); Jain *et al.* (2005 ▶); Chen *et al.* (2006 ▶). For water treatment applications, see: Izatt *et al.* (1995 ▶); Kalcher *et al.* (1995 ▶); Gilmartin & Hart (1995 ▶). For use in corrosion inhibitors, see: Ahamad *et al.* (2010 ▶); Negm *et al.* (2010 ▶). For related structures, see: Girija *et al.* (2004 ▶); Gowda *et al.* (2007 ▶)*.* For the synthesis, see: Issaadi *et al.* (2005 ▶); Ghames *et al.* (2006 ▶); Kaabi *et al.* (2007 ▶).
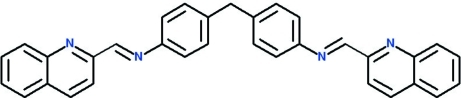


## Experimental

### 

#### Crystal data

C_33_H_24_N_4_
*M* *_r_* = 476.56
Triclinic, 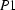

*a* = 4.6051 (2) Å
*b* = 6.0189 (2) Å
*c* = 22.2172 (8) Å
α = 88.393 (2)°
β = 88.521 (2)°
γ = 78.044 (2)°
*V* = 602.09 (4) Å^3^
*Z* = 1
Mo *K*α radiation
μ = 0.08 mm^−1^
*T* = 293 K
0.10 × 0.07 × 0.02 mm

#### Data collection

Bruker APEXII diffractometer
9094 measured reflections
2707 independent reflections
2415 reflections with *I* > 2σ(*I*)
*R*_int_ = 0.025

#### Refinement

*R*[*F* ^2^ > 2σ(*F* ^2^)] = 0.035
*wR*(*F* ^2^) = 0.087
*S* = 1.10
2707 reflections
335 parameters
3 restraints
H-atom parameters constrained
Δρ_max_ = 0.21 e Å^−3^
Δρ_min_ = −0.16 e Å^−3^

### 

Data collection: *APEX2* (Bruker, 2002 ▶); cell refinement: *SAINT* (Bruker, 2002 ▶); data reduction: *SAINT*; program(s) used to solve structure: *SHELXS97* (Sheldrick, 2008) ▶; program(s) used to refine structure: *SHELXL97* (Sheldrick, 2008) ▶; molecular graphics: *ORTEP-3 for Windows* (Farrugia, 1997 ▶); software used to prepare material for publication: *WinGX* (Farrugia, 1999 ▶).

## Supplementary Material

Crystal structure: contains datablocks I, global. DOI: 10.1107/S1600536811016011/fy2004sup1.cif

Structure factors: contains datablocks I. DOI: 10.1107/S1600536811016011/fy2004Isup2.hkl

Supplementary material file. DOI: 10.1107/S1600536811016011/fy2004Isup3.cml

Additional supplementary materials:  crystallographic information; 3D view; checkCIF report

## Figures and Tables

**Fig. 1.**
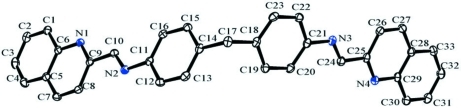
The title molecule with displacement ellipsoids for non–H atoms drawn at the 50% probability level.

## supplementary crystallographic information

### Comment

Quinolines and their derivatives are often used for the desig of synthetic compounds with diverse pharmacological and medicinal proprieties. Substituted quinolines have been reported in the literature to show antibacterial (Kidwai *et al.*, 2000), antimalarial (Souza *et al.*, 2005), antifungal (Musiol *et al.*, 2006), antiparasitical (Gómez-Barrio *et al.*, 2006), antimycobacterial (Vinsova *et al.*, 2008), antileishmanial (Jain *et al.*, 2005), and anti-inflammatory behavior (Chen *et al.*, 2006). Schiff base compounds are typically formed by condensation of an aromatic diamine and a quinolinealdehyde. These kinds of compounds have a wide variety of applications in many fields. For example, their capacity for complexation of transition metals is useful in water treatment (Izatt *et al.*, 1995; Kalcher *et al.*, 1995; Gilmartin *et al.*, 1995). They also serve as intermediates in certain enzymatic reactions and their use as corrosion inhibitors (Ahamad *et al.*, 2010; Negm *et al.*, 2010) shows their importance. The title compound, C_33_H_24_N_4_, is a condensation product of quinolinealdehyde with a bifunctional aromatic diamine. The two 4-methyl-*N*-[(*E*)-quinolin-2-ylmethylidene]aniline moieties are nearly planar. A dihedral angle of 77.86 (11)° is found between the mean planes of the benzene rings C11—C12—C13—C14—C15—C16 and C18—C19—C20—C21—C22—C23. The dihedral angle between the mean planes of the attached benzene and quinoline rings is 2.66 (9)° for the groups linked via N2 and 2.57 (9)° for those linked via N3. The corresponding bond lengths and bond angles are similar in both 4-methyl-*N*-[(*E*)-quinolin-2-ylmethylidene]aniline moieties. The N2—C10 imine (C=N) bond length of 1.270 (3) Å agrees with similar double bonds usually observed in related compounds (Girija *et al.*, 2004), and it is much shorter than the N2—C11 single C—N bond of 1.425 (2) Å (Gowda *et al.*, 2007).

### Experimental

The studied Schiff base compound was synthesized, as reported in the literature (Issaadi *et al.*, 2005; Ghames *et al.*, 2006; Kaabi *et al.*, 2007), by reacting the mixture of 4,4'-Diaminodiphenyl methane (0.396 mg, 0.002 mol) and 2-quinolinecarboxaldhyde (0.64 mg, 0.004 mol) in 20 ml of boiling ethanol for 5 h. After completion of the reaction the separated solid was filtered, washed with alcohol, and finally recrystallized from ethanol and dried under vacuum. The single crystals suitable for X-ray analysis were obtained by slow evaporation from ethanol-dichloromethane (1:1).

### Refinement

H atoms were included in geometric positions with *C*—H = 0.93 Å and *U*_iso_(H) = 1.2 *U*_eq_(C) and were refined in riding mode. In the absence of significant anomalous scattering Friedel opposites were merged.

### Crystal data

**Table:**

C_33_H_24_N_4_	*F*(000) = 250
*M**_r_* = 476.56	*D*_x_ = 1.314 Mg m^−^^3^
Triclinic, *P*1	Melting point: 472 K
*a* = 4.6051 (2) Å	Mo *K*α radiation, λ = 0.71073 Å
*b* = 6.0189 (2) Å	Cell parameters from 3977 reflections
*c* = 22.2172 (8) Å	θ = 2.8–27.4°
α = 88.393 (2)°	µ = 0.08 mm^−^^1^
β = 88.521 (2)°	*T* = 293 K
γ = 78.044 (2)°	Plate, white
*V* = 602.09 (4) Å^3^	0.10 × 0.07 × 0.02 mm
*Z* = 1	

### Data collection

**Table:**

Bruker APEXII diffractometer	2415 reflections with *I* > 2σ(*I*)
Radiation source: Enraf Nonius FR590	*R*_int_ = 0.025
graphite	θ_max_ = 27.4°, θ_min_ = 3.5°
CCD rotation images, thick slices scans	*h* = −5→5
9094 measured reflections	*k* = −7→7
2707 independent reflections	*l* = −28→28

### Refinement

**Table:**

Refinement on *F*^2^	Primary atom site location: structure-invariant direct methods
Least-squares matrix: full	Secondary atom site location: difference Fourier map
*R*[*F*^2^ > 2σ(*F*^2^)] = 0.035	Hydrogen site location: inferred from neighbouring sites
*wR*(*F*^2^) = 0.087	H-atom parameters constrained
*S* = 1.10	*w* = 1/[σ^2^(*F*_o_^2^) + (0.0373*P*)^2^ + 0.1098*P*] where *P* = (*F*_o_^2^ + 2*F*_c_^2^)/3
2707 reflections	(Δ/σ)_max_ = 0.001
335 parameters	Δρ_max_ = 0.21 e Å^−^^3^
3 restraints	Δρ_min_ = −0.16 e Å^−^^3^

### Special details

**Table:**

Geometry. All e.s.d.'s (except the e.s.d. in the dihedral angle between two l.s. planes) are estimated using the full covariance matrix. The cell e.s.d.'s are taken into account individually in the estimation of e.s.d.'s in distances, angles and torsion angles; correlations between e.s.d.'s in cell parameters are only used when they are defined by crystal symmetry. An approximate (isotropic) treatment of cell e.s.d.'s is used for estimating e.s.d.'s involving l.s. planes.
Refinement. Refinement of *F*^2^ against ALL reflections. The weighted *R*-factor *wR* and goodness of fit *S* are based on *F*^2^, conventional *R*-factors *R* are based on *F*, with *F* set to zero for negative *F*^2^. The threshold expression of *F*^2^ > σ(*F*^2^) is used only for calculating *R*-factors(gt) *etc*. and is not relevant to the choice of reflections for refinement. *R*-factors based on *F*^2^ are statistically about twice as large as those based on *F*, and *R*- factors based on ALL data will be even larger.

### Fractional atomic coordinates and isotropic or equivalent isotropic displacement parameters (Å^2^)

**Table:**

	*x*	*y*	*z*	*U*_iso_*/*U*_eq_	
N1	0.6501 (4)	0.5318 (3)	0.77370 (8)	0.0237 (4)	
N2	1.0442 (4)	0.6798 (3)	0.89610 (8)	0.0238 (4)	
N4	1.0206 (4)	−0.2145 (3)	1.38522 (9)	0.0268 (4)	
C5	0.2926 (5)	0.8583 (4)	0.73373 (10)	0.0253 (5)	
C10	0.9472 (5)	0.5623 (4)	0.85769 (10)	0.0247 (5)	
H10	1.0207	0.4063	0.8569	0.03*	
C24	1.3130 (5)	−0.1462 (4)	1.29921 (10)	0.0278 (5)	
H24	1.2005	0.0007	1.2957	0.033*	
C17	1.9310 (5)	0.3232 (4)	1.07135 (10)	0.0269 (5)	
H17A	1.9971	0.4504	1.0884	0.032*	
H17B	2.1015	0.2279	1.052	0.032*	
N3	1.5292 (4)	−0.2108 (3)	1.26316 (8)	0.0276 (4)	
C21	1.6114 (5)	−0.0656 (4)	1.21714 (10)	0.0253 (5)	
C29	0.9465 (5)	−0.3569 (4)	1.42954 (10)	0.0248 (5)	
C9	0.7206 (5)	0.6683 (4)	0.81435 (10)	0.0228 (5)	
C12	1.3477 (5)	0.7218 (4)	0.97880 (10)	0.0258 (5)	
H12	1.2586	0.8754	0.9777	0.031*	
C6	0.4393 (5)	0.6264 (4)	0.73269 (10)	0.0228 (5)	
C11	1.2656 (4)	0.5772 (4)	0.93764 (9)	0.0219 (5)	
C25	1.2375 (5)	−0.3021 (4)	1.34684 (10)	0.0258 (5)	
C16	1.4071 (5)	0.3483 (4)	0.93972 (10)	0.0251 (5)	
H16	1.3565	0.2488	0.9125	0.03*	
C28	1.0849 (5)	−0.5901 (4)	1.43483 (10)	0.0265 (5)	
C15	1.6231 (5)	0.2676 (4)	0.98216 (10)	0.0250 (5)	
H15	1.7168	0.1151	0.9826	0.03*	
C22	1.8462 (5)	−0.1645 (4)	1.17962 (10)	0.0272 (5)	
H22	1.9367	−0.3162	1.1859	0.033*	
C4	0.0736 (5)	0.9418 (4)	0.69045 (11)	0.0327 (5)	
H4	−0.0249	1.0932	0.6911	0.039*	
C7	0.3754 (5)	0.9952 (4)	0.77817 (10)	0.0296 (5)	
H7	0.2831	1.1476	0.7804	0.035*	
C8	0.5909 (5)	0.9030 (4)	0.81772 (10)	0.0267 (5)	
H8	0.6519	0.9923	0.8464	0.032*	
C13	1.5611 (5)	0.6402 (4)	1.02157 (10)	0.0262 (5)	
H13	1.611	0.7397	1.0489	0.031*	
C33	0.9868 (5)	−0.7265 (4)	1.48092 (11)	0.0309 (5)	
H33	1.0742	−0.88	1.4843	0.037*	
C32	0.7644 (6)	−0.6344 (4)	1.52053 (11)	0.0340 (6)	
H32	0.702	−0.7254	1.5506	0.041*	
C30	0.7182 (5)	−0.2664 (4)	1.47154 (11)	0.0301 (5)	
H30	0.6277	−0.1134	1.4689	0.036*	
C1	0.3662 (5)	0.4863 (4)	0.68751 (10)	0.0272 (5)	
H1	0.4637	0.3349	0.6857	0.033*	
C14	1.7009 (5)	0.4130 (4)	1.02415 (10)	0.0237 (5)	
C19	1.5822 (5)	0.2860 (4)	1.15999 (10)	0.0273 (5)	
H19	1.4927	0.438	1.1537	0.033*	
C23	1.9483 (5)	−0.0403 (4)	1.13279 (11)	0.0286 (5)	
H23	2.1062	−0.1098	1.1083	0.034*	
C3	0.0056 (5)	0.8029 (5)	0.64793 (12)	0.0359 (6)	
H3	−0.1385	0.8601	0.6198	0.043*	
C2	0.1522 (5)	0.5731 (4)	0.64641 (11)	0.0322 (5)	
H2	0.1037	0.4795	0.6173	0.039*	
C18	1.8167 (5)	0.1865 (4)	1.12220 (10)	0.0240 (5)	
C27	1.3166 (5)	−0.6745 (4)	1.39298 (10)	0.0304 (5)	
H27	1.4141	−0.826	1.3951	0.036*	
C26	1.3957 (5)	−0.5313 (4)	1.34953 (10)	0.0287 (5)	
H26	1.5499	−0.583	1.3223	0.034*	
C20	1.4791 (5)	0.1629 (4)	1.20683 (10)	0.0276 (5)	
H20	1.3218	0.2326	1.2314	0.033*	
C31	0.6299 (5)	−0.4020 (4)	1.51582 (11)	0.0333 (5)	
H31	0.4799	−0.3404	1.543	0.04*	

### Atomic displacement parameters (Å^2^)

**Table:**

	*U*^11^	*U*^22^	*U*^33^	*U*^12^	*U*^13^	*U*^23^
N1	0.0219 (9)	0.0260 (9)	0.0232 (9)	−0.0050 (8)	0.0006 (7)	−0.0012 (7)
N2	0.0213 (9)	0.0274 (10)	0.0233 (9)	−0.0064 (8)	0.0020 (7)	−0.0009 (7)
N4	0.0286 (10)	0.0285 (10)	0.0242 (10)	−0.0084 (8)	−0.0018 (8)	0.0014 (8)
C5	0.0194 (11)	0.0292 (12)	0.0257 (11)	−0.0026 (9)	0.0053 (9)	0.0032 (9)
C10	0.0232 (11)	0.0251 (11)	0.0245 (11)	−0.0025 (9)	0.0016 (9)	−0.0010 (9)
C24	0.0323 (13)	0.0281 (11)	0.0241 (11)	−0.0085 (10)	−0.0032 (10)	0.0005 (9)
C17	0.0204 (11)	0.0363 (13)	0.0259 (11)	−0.0107 (10)	−0.0012 (9)	0.0023 (10)
N3	0.0275 (11)	0.0335 (11)	0.0222 (10)	−0.0072 (9)	−0.0013 (8)	0.0007 (8)
C21	0.0258 (12)	0.0319 (12)	0.0203 (11)	−0.0097 (10)	−0.0038 (9)	−0.0018 (9)
C29	0.0239 (11)	0.0304 (12)	0.0217 (11)	−0.0090 (9)	−0.0054 (9)	0.0014 (9)
C9	0.0195 (11)	0.0278 (12)	0.0210 (10)	−0.0051 (9)	0.0048 (9)	0.0007 (9)
C12	0.0229 (11)	0.0246 (11)	0.0301 (12)	−0.0055 (9)	0.0025 (9)	−0.0042 (9)
C6	0.0185 (11)	0.0269 (12)	0.0230 (11)	−0.0056 (9)	0.0055 (8)	0.0025 (9)
C11	0.0196 (11)	0.0276 (12)	0.0196 (10)	−0.0079 (9)	0.0028 (8)	−0.0010 (9)
C25	0.0299 (12)	0.0303 (12)	0.0195 (10)	−0.0109 (10)	−0.0045 (9)	0.0002 (9)
C16	0.0258 (12)	0.0281 (12)	0.0225 (11)	−0.0074 (9)	0.0016 (9)	−0.0052 (9)
C28	0.0286 (12)	0.0291 (12)	0.0240 (11)	−0.0101 (9)	−0.0066 (9)	0.0000 (9)
C15	0.0222 (11)	0.0259 (11)	0.0265 (11)	−0.0038 (9)	0.0006 (9)	−0.0007 (9)
C22	0.0261 (12)	0.0281 (12)	0.0265 (12)	−0.0036 (9)	−0.0007 (10)	−0.0017 (9)
C4	0.0241 (12)	0.0336 (13)	0.0367 (13)	0.0010 (10)	0.0018 (10)	0.0072 (10)
C7	0.0297 (13)	0.0248 (12)	0.0313 (12)	0.0001 (10)	0.0067 (10)	−0.0004 (9)
C8	0.0301 (12)	0.0265 (11)	0.0229 (11)	−0.0043 (9)	0.0038 (9)	−0.0046 (9)
C13	0.0238 (11)	0.0331 (13)	0.0239 (11)	−0.0105 (10)	0.0005 (9)	−0.0055 (9)
C33	0.0335 (14)	0.0297 (12)	0.0311 (13)	−0.0101 (11)	−0.0069 (10)	0.0057 (10)
C32	0.0382 (14)	0.0379 (13)	0.0296 (12)	−0.0175 (11)	−0.0027 (10)	0.0092 (10)
C30	0.0290 (12)	0.0337 (12)	0.0283 (11)	−0.0075 (10)	−0.0024 (9)	0.0005 (9)
C1	0.0253 (11)	0.0307 (12)	0.0259 (11)	−0.0072 (9)	0.0015 (9)	0.0008 (9)
C14	0.0170 (10)	0.0346 (13)	0.0210 (10)	−0.0094 (9)	0.0046 (8)	0.0017 (9)
C19	0.0260 (12)	0.0300 (12)	0.0258 (11)	−0.0060 (9)	−0.0024 (9)	0.0015 (9)
C23	0.0233 (11)	0.0365 (13)	0.0263 (11)	−0.0063 (10)	0.0040 (9)	−0.0075 (10)
C3	0.0251 (12)	0.0507 (16)	0.0315 (12)	−0.0080 (11)	−0.0064 (10)	0.0125 (11)
C2	0.0289 (12)	0.0443 (14)	0.0262 (12)	−0.0145 (11)	−0.0013 (9)	0.0013 (10)
C18	0.0175 (10)	0.0363 (12)	0.0201 (10)	−0.0098 (9)	−0.0032 (8)	−0.0013 (9)
C27	0.0349 (13)	0.0259 (11)	0.0294 (12)	−0.0033 (10)	−0.0062 (10)	−0.0006 (9)
C26	0.0315 (12)	0.0321 (12)	0.0218 (10)	−0.0049 (10)	−0.0005 (9)	−0.0030 (9)
C20	0.0240 (11)	0.0347 (12)	0.0233 (11)	−0.0043 (9)	0.0020 (9)	−0.0021 (9)
C31	0.0302 (13)	0.0433 (14)	0.0283 (12)	−0.0124 (11)	0.0005 (10)	−0.0003 (10)

### Geometric parameters (Å, °)

**Table:**

N1—C9	1.328 (3)	C28—C33	1.418 (3)
N1—C6	1.373 (3)	C15—C14	1.399 (3)
N2—C10	1.270 (3)	C15—H15	0.93
N2—C11	1.424 (3)	C22—C23	1.393 (3)
N4—C25	1.329 (3)	C22—H22	0.93
N4—C29	1.370 (3)	C4—C3	1.363 (4)
C5—C7	1.412 (3)	C4—H4	0.93
C5—C4	1.417 (3)	C7—C8	1.362 (3)
C5—C6	1.420 (3)	C7—H7	0.93
C10—C9	1.471 (3)	C8—H8	0.93
C10—H10	0.93	C13—C14	1.386 (3)
C24—N3	1.265 (3)	C13—H13	0.93
C24—C25	1.477 (3)	C33—C32	1.370 (4)
C24—H24	0.93	C33—H33	0.93
C17—C14	1.517 (3)	C32—C31	1.409 (4)
C17—C18	1.526 (3)	C32—H32	0.93
C17—H17A	0.97	C30—C31	1.368 (3)
C17—H17B	0.97	C30—H30	0.93
N3—C21	1.421 (3)	C1—C2	1.373 (3)
C21—C22	1.390 (3)	C1—H1	0.93
C21—C20	1.399 (3)	C19—C20	1.391 (3)
C29—C30	1.419 (3)	C19—C18	1.394 (3)
C29—C28	1.419 (3)	C19—H19	0.93
C9—C8	1.418 (3)	C23—C18	1.390 (3)
C12—C13	1.390 (3)	C23—H23	0.93
C12—C11	1.392 (3)	C3—C2	1.408 (4)
C12—H12	0.93	C3—H3	0.93
C6—C1	1.419 (3)	C2—H2	0.93
C11—C16	1.397 (3)	C27—C26	1.368 (3)
C25—C26	1.421 (3)	C27—H27	0.93
C16—C15	1.392 (3)	C26—H26	0.93
C16—H16	0.93	C20—H20	0.93
C28—C27	1.418 (3)	C31—H31	0.93
			
C9—N1—C6	117.22 (19)	C3—C4—H4	119.6
C10—N2—C11	121.13 (17)	C5—C4—H4	119.6
C25—N4—C29	117.13 (19)	C8—C7—C5	119.7 (2)
C7—C5—C4	123.2 (2)	C8—C7—H7	120.1
C7—C5—C6	117.7 (2)	C5—C7—H7	120.1
C4—C5—C6	119.1 (2)	C7—C8—C9	118.8 (2)
N2—C10—C9	121.21 (18)	C7—C8—H8	120.6
N2—C10—H10	119.4	C9—C8—H8	120.6
C9—C10—H10	119.4	C14—C13—C12	121.1 (2)
N3—C24—C25	120.6 (2)	C14—C13—H13	119.4
N3—C24—H24	119.7	C12—C13—H13	119.4
C25—C24—H24	119.7	C32—C33—C28	120.6 (2)
C14—C17—C18	113.55 (17)	C32—C33—H33	119.7
C14—C17—H17A	108.9	C28—C33—H33	119.7
C18—C17—H17A	108.9	C33—C32—C31	120.2 (2)
C14—C17—H17B	108.9	C33—C32—H32	119.9
C18—C17—H17B	108.9	C31—C32—H32	119.9
H17A—C17—H17B	107.7	C31—C30—C29	120.7 (2)
C24—N3—C21	122.4 (2)	C31—C30—H30	119.7
C22—C21—C20	118.4 (2)	C29—C30—H30	119.7
C22—C21—N3	115.5 (2)	C2—C1—C6	120.3 (2)
C20—C21—N3	126.1 (2)	C2—C1—H1	119.8
N4—C29—C30	118.1 (2)	C6—C1—H1	119.8
N4—C29—C28	123.1 (2)	C13—C14—C15	118.2 (2)
C30—C29—C28	118.8 (2)	C13—C14—C17	121.1 (2)
N1—C9—C8	124.0 (2)	C15—C14—C17	120.7 (2)
N1—C9—C10	116.01 (18)	C20—C19—C18	121.4 (2)
C8—C9—C10	120.03 (18)	C20—C19—H19	119.3
C13—C12—C11	121.0 (2)	C18—C19—H19	119.3
C13—C12—H12	119.5	C18—C23—C22	120.7 (2)
C11—C12—H12	119.5	C18—C23—H23	119.7
N1—C6—C5	122.56 (19)	C22—C23—H23	119.7
N1—C6—C1	118.5 (2)	C4—C3—C2	120.4 (2)
C5—C6—C1	118.9 (2)	C4—C3—H3	119.8
C12—C11—C16	118.22 (19)	C2—C3—H3	119.8
C12—C11—N2	115.96 (19)	C1—C2—C3	120.5 (2)
C16—C11—N2	125.81 (18)	C1—C2—H2	119.7
N4—C25—C26	124.0 (2)	C3—C2—H2	119.7
N4—C25—C24	116.30 (19)	C23—C18—C19	118.2 (2)
C26—C25—C24	119.7 (2)	C23—C18—C17	120.7 (2)
C15—C16—C11	120.7 (2)	C19—C18—C17	121.1 (2)
C15—C16—H16	119.7	C26—C27—C28	119.5 (2)
C11—C16—H16	119.7	C26—C27—H27	120.2
C27—C28—C33	123.3 (2)	C28—C27—H27	120.2
C27—C28—C29	117.5 (2)	C27—C26—C25	118.7 (2)
C33—C28—C29	119.2 (2)	C27—C26—H26	120.6
C16—C15—C14	120.9 (2)	C25—C26—H26	120.6
C16—C15—H15	119.6	C19—C20—C21	120.2 (2)
C14—C15—H15	119.6	C19—C20—H20	119.9
C21—C22—C23	121.1 (2)	C21—C20—H20	119.9
C21—C22—H22	119.4	C30—C31—C32	120.5 (2)
C23—C22—H22	119.4	C30—C31—H31	119.7
C3—C4—C5	120.8 (2)	C32—C31—H31	119.7
